# Visual functions and multimodal imaging of patients with idiopathic focal choroidal excavation

**DOI:** 10.1038/s41598-024-63866-3

**Published:** 2024-06-09

**Authors:** Akiko Okubo, Fumiki Okamoto, Kei Uezono, Kazuhiko Unoki

**Affiliations:** 1 Unoki Eye Clinic, Harara 1-7-15, Kagoshima City, Kagoshima, 890-0026 Japan; 2 Department of Ophthalmology, Graduate School of Medicine, Nippon Medical School, Tokyo, Japan

**Keywords:** Idiopathic focal choroidal excavation, Visual function, Metamorphopsia, Retinal diseases, Vision disorders

## Abstract

This study aimed to evaluate visual function and perform multimodal imaging on patients with focal choroidal excavation without any chorioretinal disease (idiopathic focal choroidal excavation [iFCE]). Seventeen eyes of 15 patients with iFCE (8 men, 7 women; mean ± standard deviation age, 56.0 ± 10.8 years) were assessed for visual function including visual acuity, metamorphopsia, aniseikonia, and retinal sensitivity. Multimodal imaging included optical coherence tomography (OCT), fundus autofluorescence (FAF), and OCT angiography. This study found that the maximum width and depth of the excavation were 597 ± 330 (238–1809) µm and 123 ± 45 (66–231) µm, respectively, and that FAF showed normal or hypoautofluorescence corresponding to iFCE. The fundus examination findings were stable during the follow-up period (96 ± 48 months). None of the eyes showed any abnormalities in central retinal sensitivity or aniseikonia. Metamorphopsia was detected using Amsler grid testing and M-CHARTS in two eyes. Therefore, this study is the first to quantitatively and qualitatively study metamorphopsia of patients with iFCE. Our results showed that most patients with iFCE did not have visual impairments, despite the presence of morphological changes in the outer retina and choroid.

## Introduction

Focal choroidal excavation (FCE) is defined as an area of concavity in the choroid without accompanying scleral ectasia or posterior staphyloma in the macular and extra-macular regions and is a relatively rare condition^1,2^. FCE is detected using optical coherence tomography (OCT) and is commonly subdivided based on the presence (non-conforming) or absence (conforming) of a separation between the photoreceptor tips and the retinal pigment epithelium (RPE) morphologically^2^. Most cases of FCE are diagnosed when the patient is around 40–50 years old, although cases occurring in patients of all ages have been reported. Early investigators postulated that FCE is congenital^1–3^; however, acquired FCE is increasingly being recognized via longitudinal analyses and associations with various chorioretinal diseases, such as central serous chorioretinopathy (CSC), choroidal neovascularization, polypoidal choroidal vasculopathy, uveitis, macular dystrophy, and tumors^4–17^. Additionally, the association between FCE and pachychoroid spectrum disorders is recognized^16,18^. Hence, a classification based on etiology has been proposed^19^. Acquired FCEs are acquired after birth with chorioretinal disease development and show visual symptoms, such as blurring of vision and metamorphopsia, that depend on the underlying chorioretinal diseases. Congenital FCEs show no evidence of chorioretinal disease, and most are incidentally found without any visual symptoms^3,7,16,17,20^. That said, some patients reportedly do have visual symptoms^3,7^, although the details have not been described.

Accordingly, the present study aimed to clarify these details by examining the visual functions and clinical features, including visual acuity, metamorphopsia, retinal sensitivity, aniseikonia, and multimodal imaging, of patients with the congenital type of FCE, termed idiopathic FCE (iFCE).

## Results

### Clinical characteristics

Seventeen eyes of 15 patients with iFCE were included in this study. The clinical characteristics, visual functions, and retinal microstructures of patients with iFCE are shown in Table 1. Among the 15 patients, 8 were men and 7 were women, with a mean ± standard deviation (range) age of 56.0 ± 10.8 (34–73) years (Table 2). Thirteen patients had unilateral involvement and two patients had bilateral involvement (patient numbers 7 and 14). Fifteen eyes had one excavation, one eye had two excavations, and one eye had three excavations. All eyes were phakic, and the grade of the cataracts was not severe enough to threaten visual acuity. Fifteen eyes were myopic, and the refractive error of the involved eyes was − 3.72 ± 2.44 (− 7.50– + 1.75) diopters (D). The follow-up period after iFCE was recorded using OCT was 96 ± 48 (range, 1–155) months. In one patient, the fellow eye exhibited a retinochoroidal scar due to ocular toxoplasmosis. In another patient, the fellow eye showed exudative age-related macular degeneration (AMD) and was treated with an anti-vascular endothelial growth factor drug. The FCEs in three patients were detected by chance during follow-up in our Glaucoma Outpatient Clinic. In one patient with abducens nerve palsy, FCE was detected in the same eye with the palsy. Only one patient visited us with metamorphopsia (patient number 7). In the others, macular pigment abnormality was pointed out by medical checkup or at the other outpatient clinics in our institution. They were introduced to the Medical Retinal Outpatient Clinic resulting in the detection of FCEs.

**Table 1:** Clinical characteristics, visual functions and retinal microstructure in patients with idiopathic focal choroidal excavation.

Patient	Sex	Age (years)	BCVA (logMAR)	M- CHARTS (MV/MH)	Number of FCE	Location of FCE	Fundus finding of FCE	FAF	Maximum width of FCE (μm)	Maximum depth of FCE (μm)	Foveal choroidal thickness (μm)
1	F	59	0.00	0/0	1	SF	whitish	n.p	656	140	141
2	M	56	0.05	0/0	1	SF	whitish	hyper	640	143	301
3	F	54	0.00	0/0	1	EF	whitish	hypo	464	132	248
4	M	69	0.00	0/0	1	SF	orange	n.p	434	231	188
5	F	61	− 0.08	0/0	1	SF	orange	n.p	556	136	109
6	M	65	− 0.08	0/0.3	1	SF	whitish	hypo	561	154	179
7	F	44	− 0.08	0/0	2	EF × 2	whitish	hypo × 2	618/238	73/71	315
			− 0.18	0.3/0.5	1	SF	whitish	hypo	1809	218	318
8	M	73	0.00	0/0	1	EF	whitish	n.p	369	108	219
9	M	56	0.05	0/0	1	SF	orange	n.p	933	156	212
10	M	63	0.05	0/0	1	EF	normal	n.p	284	79	358
11	M	34	− 0.08	0/0	1	SF	whitish	n.p	619	127	298
12	F	63	0.00	0/0	3	EF × 3	whitish, orange × 2	hypo × 2, n.p	589/473/437	70/95/66	400
13	F	42	− 0.08	0/0	1	EF	orange	n.p	333	114	254
14	M	56	− 0.08	0/0	1	EF	whitish	hypo	775	125	245
			− 0.08	0/0	1	EF	whitish	hypo	664	120	270
15	F	45	− 0.08	0/0	1	EF	whitish	hypo	490	111	148

*FCE* focal choroidal excavation, *F* female, *M* male, *BCVA* best corrected visual acuity, *logMAR* logarithm of the minimum angle of resolution, *MV* vertical metamorphopsia, *MH* horizontal metamorphopsia, *SF* subfovea, *EF* extrafovea, *FAF* fundus autofluorescence, *hypo* hypoautofluorescence, *hyper* hyperautofluorescence, *n.p.* = nothing particular.

**Table 2:** Literature review of the clinical feature of idiopathic focal choroidal excavation.

	Wakabayashi^3^	Shinojima^7^	Chung^16^	Gan^17^	Szabelska^20^	Present study
Patient demographics						
Number of eyes (patients)	3 (3)	7 (7)	9 (9)	5 (4)	13 (13)	17 (15)
Mean age (range)	35 (33–38)	50 (30–72)	48 (33–69)	37 (± 15)	(23–64)	56 (34–73)
Sex (male/female)	0/3	5/2	4/5	N/A	1/12	8/7
Race	Japanese	Japanese	N/A	Chinese	Caucasian	Japanese
Mean spherical equivalent (range)	− 7.0 D (− 8D-0D)	− 4.79D (− 7.63D ~ − 1.75D)	(− 9.25D ~ + 7.0D)	− 1.5D (− 6D ~ 0D)	(− 4.5D ~ − 3.0D)*	− 3.72D (− 7.5D ~ + 1.75D)
Location of FCE (extrafovea/subfovea)	0/3	N/A	2/7	N/A	3/11	12/8
Visual functions						
Mean best corrected visual acuity (logMAR)	− 0.12	− 0.04	0.03	0	(range, 0–0.15)*	− 0.04
Frequency of having metamorphopsia (%)	100	N/A	N/A	N/A	N/A	12
Central visual field	all normal	N/A	N/A	N/A	N/A	all normal
Aniseikonia	N/A	N/A	N/A	N/A	N/A	None
Imaging						
Classification (conforming /non-conforming)	1/2	N/A	7/2	6/0	13/1	19/1
Mean foveal choroidal thickness (urn)	N/A	304	N/A	289	N/A	247
Fundus autofluorescence	hypo	N/A	hypo, hyper	N/A	hypo	normal, hypo
Clinical course	stable	stable	stable	stable	stable	stable

*FCE* focal choroidal excavation, *N/A* not available, *D* diopter, *hypo* hypoautofluorescence, *hyper* hyperautofluorescence, *data includes one patient with secondary neovascularization.

### Multimodal imaging

Fundus examination showed no abnormalities other than pigmentary disturbances corresponding to FCE. Pigmentary disturbances included a depigmented or whitish appearance in 12 of the 20 FCEs. In six FCEs, the orange color, possibly originating from the choroidal vessels, was remarkable. Eight FCEs involved the fovea, whereas the others were located extrafoveally. During the follow-up period, fundus findings were stable, without the development of any retinochoroidal disease in any eye.

Based on the morphological classification, which was whether there was a separation between the photoreceptor and the RPE, one FCE (patient number 4) was the non-confirming type (FCE with separation) and the others were the conforming type (FCE with no separation). Mutual conversion of the two types was not observed in any of the eyes. The bands of the external limiting membrane and ellipsoid zone were preserved in all eyes. The refractive bands originating from the outer segment tips were indistinguishable in 19 of the 20 FCEs. Although the line corresponding to the RPE within the excavation was preserved in 19 FCEs, some parts of it were irregular in one FCE. The outer nuclear layer and/or outer plexiform layer were partially thick, except in two eyes. The mean ± standard deviation (range) maximum width and depth of the excavation were 597 ± 330 (238–1809) µm and 123 ± 45 (66–231) µm, respectively. The subfoveal choroidal thickness was 247 ± 80 (109–400) µm.

Regarding the fundus autofluorescence (FAF), it was difficult to judge whether the results showed hypoautofluorescence or normal findings when the FCEs were located in the fovea, since the foveal center exhibited hypoautofluorescence in the blue FAF due to normal absorption of blue light by the luteal pigment and melanin^21,22^. We labeled two FCEs of two patients as demonstrating hypoautofluorescence, given that the hypoautofluorescence areas were consistent with the areas of the excavation. Hypoautofluorescence was observed in 8 out of 12 extrafoveal FCEs, while its degree varied from slight to obvious. One FCE revealed a hyperautofluorescence spot that may have originated from a large druse (pachydruse) within the area of the FCE.

### Visual functions

The mean ± standard deviation (range) best-corrected visual acuity (BCVA) was − 0.04 ± 0.06 (− 0.18–0.05) logarithm of the minimum angle of resolution (logMAR) (Table 2). No abnormalities were detected in relation to the FCE lesions using the HFA 10–2 test, and none of the eyes showed aniseikonia using the New Aniseikonia Test (NAT). Two of the 17 eyes (12%) revealed metamorphopsia on both M-CHARTS and Amsler grid testing. In one patient with subfoveal FCE who had not been aware of metamorphopsia before the examinations, the horizontal and vertical metamorphopsia M-CHARTS scores were 0 and 0.3, respectively, and distortion was detected just temporal to the center using Amsler grid testing. Relationships among metamorphopsia, subfoveal choroidal thickness, and excavation size were not observed.

### Representative cases

#### FCE without metamorphopsia, conforming type (Patient Number 9)

**Figure 1:**
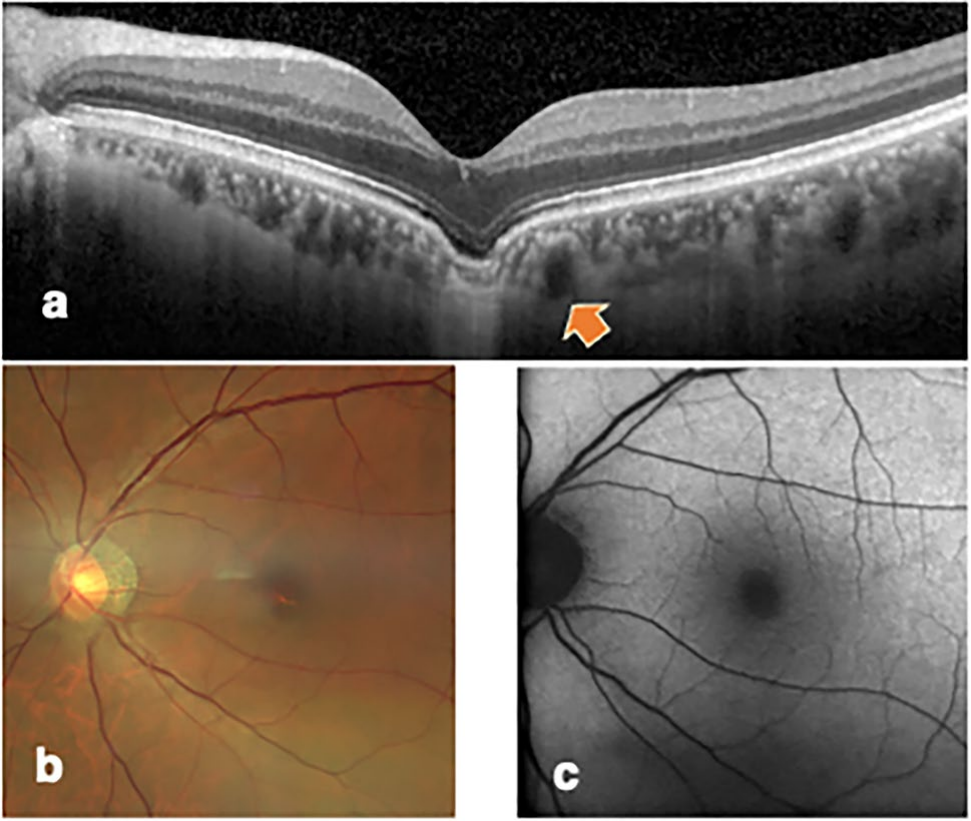
Images of the left eye of a 56-year-old man (patient number 9) with a conforming focal choroidal excavation of the left eye. The best-corrected visual acuity was 0.05 the logarithm of the minimum angle of resolution. (**a**) Enhanced depth imaging spectral domain optical coherent tomography centered on the fovea horizontally (from nasal to temporal) revealed a subfoveal choroidal excavation with a maximum width and depth of 933 µm and 156 µm, respectively, in the left eye. A pachychoroid vessel (arrow) was observed near the excavation site. (**b**) Color fundus photograph showed no abnormalities, except the orange color, originating from the choroidal vessels, was remarkable at the fovea. (**c**) Fundus autofluorescence did not show any abnormalities. No abnormalities were detected using the Humphrey Field Analyzer 10–2 program. The results of the Amsler grid testing, M-CHARTS, and New Aniseikonia Test were normal.

A 56-year-old man presented to our clinic for eye examinations. His BCVA and refraction was 0.00 logMAR and − 6.50 D, respectively, in the right eye, and 0.05 logMAR and − 6.50 D, respectively, in the left eye. OCT revealed a subfoveal choroidal excavation with a maximum width and depth of 933 µm and 156 µm, respectively, in the left eye. The left fovea was orange in color. No abnormalities were detected in the central visual field test, Amsler grid test, M-CHARTS, or NAT. During the follow-up period of 2 years, his visual acuity and fundus examination findings were stable in both eyes (Fig. 1).

#### FCE without metamorphopsia, non-conforming type (Patient Number 4)

**Figure 2:**
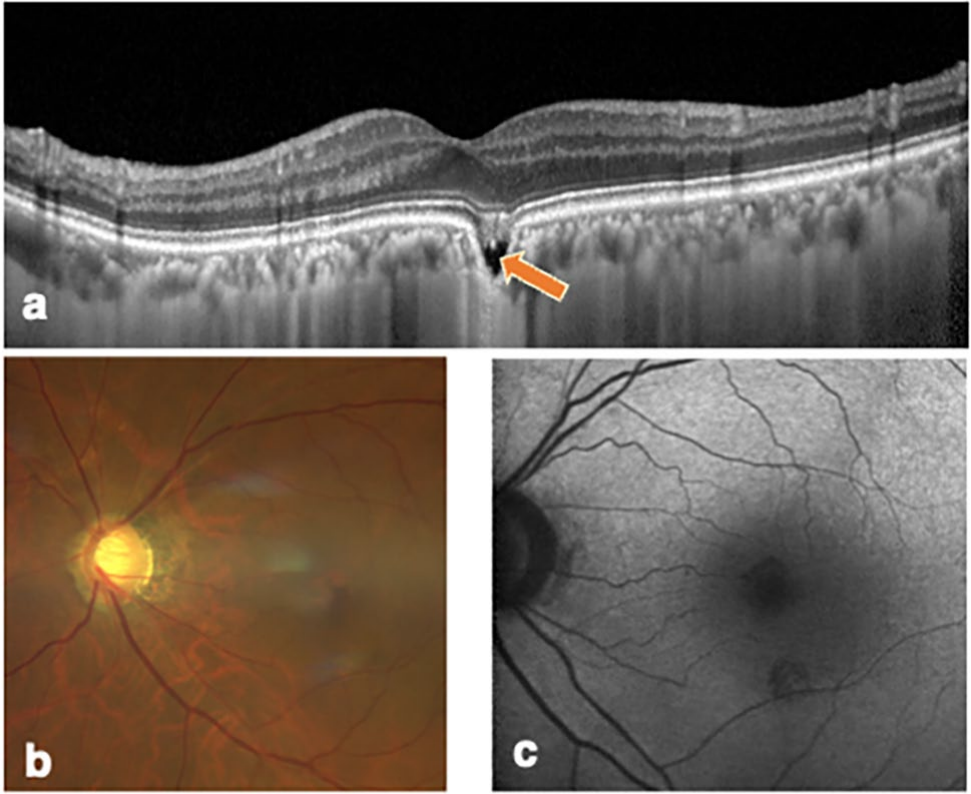
Images of the left eye of a 69-year-old man (patient number 4) with a non-conforming focal choroidal excavation. The best-corrected visual acuity was 0.00, the logarithm of the minimum angle of resolution. (**a**) Enhanced depth imaging spectral domain optical coherent tomography vertically centered on the fovea (from inferior to superior) revealed a choroidal excavation just superior to the fovea with a maximum width and depth of 434 µm and 231 µm, respectively, in the left eye. There was a separation (arrow) between the photoreceptor tips and the retinal pigment epithelium. Hyperreflective material was seen in the separated space. (**b**) Color fundus photograph showing an orange color in the fovea, probably originating from the choroidal vessels. (**c**) Fundus autofluorescence did not show any abnormalities corresponding to the FCE. No abnormalities were detected using the Humphrey Field Analyzer 10–2 program. The results of the Amsler grid testing, M-CHARTS, and the New Aniseikonia Test were normal.

A 69-year-old man was introduced to our clinic due to FCE that was incidentally detected during follow-up for primary open-angle glaucoma. His BCVA and refraction was 0.00 logMAR and − 7.50 D, respectively in both eyes. Optical coherence tomography revealed a choroidal excavation with a maximum width and depth of 434 µm and 231 µm, respectively, in the left eye. The left fovea was orange in color. No abnormalities were detected in the central visual field test, Amsler grid test, M-CHARTS, or NAT. During the follow-up period of 4.5 years, his visual acuity and fundus examination findings were stable in both eyes (Fig. 2).

#### FCE with Metamorphopsia (Patient Number 7)

**Figure 3:**
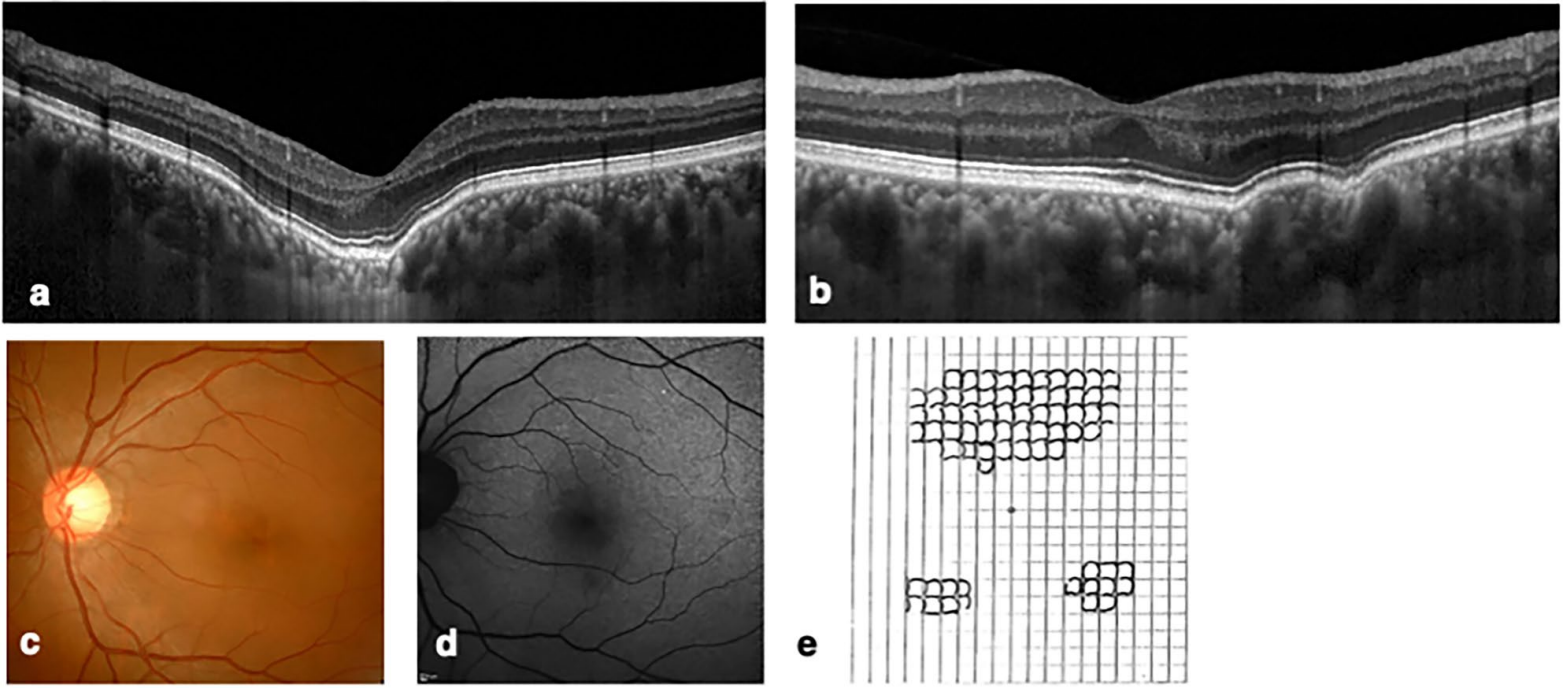
Images of the eyes of a 44-year-old woman (patient number 7) with bilateral focal choroidal excavations. The best-corrected visual acuity (logarithm of the minimum angle of resolution) was − 0.08 in the right eye and − 0.18 in the left eye. (**a**) and (**b**) Enhanced depth imaging spectral domain optical coherent tomography centered on the fovea vertically (from inferior to superior) showed a subfoveal choroidal excavation in the left eye (**a**) and two extrafoveal excavations in the right eye (**b**). (**c**) Color fundus photograph of the left eye showed an orange appearance in the foveal area. (**d**) Fundus autofluorescence of the left eye demonstrated hypoautofluorescence corresponding to the excavation area. (**e**) Amsler grid test of the left eye, wherein the central four squares were fine but the grid lines in the other parts, especially superior quadrants to the center of the grid looked like “precisely handwritten lines rather than printed lines” per her report. Horizontal and vertical scores of metamorphopsia using M-CHARTS were 0.5 and 0.3, respectively. During the follow-up period, her visual acuity and fundus examination findings were stable in both eyes.

A 44-year-old woman presented to our clinic with metamorphopsia in the left eye that started 7 months prior to the first clinical visit. Her BCVA and refraction was − 0.08 logMAR and − 3.00 D, respectively, in the right eye, and − 0.18 logMAR and − 4.25 D, respectively, in the left eye. Fundus examination findings were normal, except for a reddish or depigmented macula in both eyes. OCT revealed two extrafoveal excavations in the right eye and a subfoveal choroidal excavation in the left eye. Areas corresponding to FCEs showed slight fundus hypoautofluorescence in both eyes. Amsler grid testing confirmed metamorphopsia in the left eye, with M-CHARTS horizontal and vertical scores of 0.5 and 0.3, respectively. No abnormalities were detected in the central visual field test or NAT. During the follow-up period of 8.5 years, her visual acuity and fundus examination findings were stable in both eyes (Fig. 3).

## Discussion

We explored the clinical features and assessed the visual function, including metamorphopsia, of patients with iFCE. Our results revealed that patients with iFCE showed no sex differences and myopic tendencies. Additionally, they had fewer bilateral lesions. These results were consistent with the results of 37 eyes with iFCE from five previously reported studies (Table 2)^3,7,16,17,20^, wherein the data of each patient could be available and some patients were confirmed as having probable idiopathic FCE. On the contrary, there were less incidents of myopia in eyes with both FCE and AMD^6^ but more bilateral lesions in the cohort with FCE and AMD/CSC^5,6,11^. These results suggest that the refraction and bilateral/unilateral involvement are influenced by the original characteristics of the coexisting diseases in patients with acquired FCE. The mean age of the patients in the present study was higher than those in the other five studies, probably due to our patients were sourced from the Medical Retina Outpatient Clinic, wherein most of the patients who visited had age-related eye diseases. Nevertheless, the age range was consistent with those obtained in the other four studies^3,7,16,17^. The mean subfoveal choroidal thickness of the eyes in this study (247 µm) was thinner than those of the two studies by Shinojima et al.^7^ (304 µm) and Gan et al.^17^ (289 µm). These values in patients with iFCEs were not substantially different from those in patients with acquired FCEs^4–7,15^.

Fundus examination findings corresponding to iFCEs, including pigmentary disturbances, such as mottling and hypo- and hyperpigmentation, have been reported^3,7,16,17,20^. An orange appearance within or near the FCEs was noted in six FCE lesions of this study. This probably originates from the underlying or adjacent large choroidal vessels in Haller’s layer, which was at the level of the bottom of the excavation in most of our cases. This may support the recently proposed concept that FCE is one of the manifestations of pachychoroid spectrum disorders, a group of entities that share common phenotypic features such as increased choroidal thickness and dilation of the Haller’s layer vessels (pachyvessels) which compress the overlying choriocapillaris and Sattler’s layer^16,18^. For FAF, hypo- and hyperautofluorescence, or normal appearance, have been reported as corresponding to iFCEs^3,16,19,20^. In our study, 10 of 20 FCEs (50%) showed hypoautofluorescence, while the others showed no abnormalities. Because the dominant source of the FAF signal is light excitation of the fluorophores in lipofuscin at the level of the RPE^21,23^, irregular shapes of the concavities may lead to a thinner or irregular RPE, which may result in hypoautofluorescence.

In this study, none of the eyes showed any abnormalities with respect to central retinal sensitivity or aniseikonia, which has not been examined previously in patients with iFCE. The mean BCVA of patients with iFCE of − 0.04 logMAR in the current study was similar to those in the other five reports^3,7,16,17,20^. Metamorphopsia, which was not quantified in the other studies, was detected herein using both Amsler grid testing and M-CHARTS and was identified in two patients. Therefore, the present study is the first to quantitatively and qualitatively study metamorphopsia using M-CHART and Amsler grid testing, respectively. Our results showed that most patients did not have visual impairments, although two patients exhibited metamorphopsia, as mentioned above. One of our patients (patient number 7) stated that she had clearly noticed metamorphopsia 7 months before the first clinical visit and that the symptoms had become less severe. Interestingly, the three patients in the study by Wakabayashi et al.^3^ noticed metamorphopsia 2 weeks, 7 months, and 1 year, respectively, prior to the examination. Although we called our cases idiopathic, some of the patients might have had a previous history of eye diseases that was not declared or could not be inferred from their clinical findings. For example, FCE was reported to be present in 7.8% of eyes with CSC, which is known as a self-limiting disease^5^. Data on metamorphopsia in eyes with recovered CSC and FCE are not currently available. Regarding metamorphopsia in patients with CSC, Fujita et al.^24^ studied the change from before photodynamic therapy to 1 year after the complete resolution of serous retinal detachment using M-CHARTS and showed that the metamorphopsia improved after treatment, but it was still persistent. Therefore, it is possible that patients with FCE and recovered CSC might also exhibit metamorphopsia.

Park and Oh^25^ reported that FCE was detected in only 3 out of 1697 eyes (0.18%) from children and young adults (range, 3–39 years). The authors concluded that the extremely low prevalence of FCE in the cohort may suggest that the congenital type of FCE is rare, and the majority of FCE found in adults is most likely acquired. Chung et al.^16^ postulated that focal choroidal atrophy, unrelated to a congenital developmental defect but instead due to subclinical choroidal inflammation or choroidal ischemia, could be a mechanism leading to FCE formation in the absence of scarring. Ellabban et al.^5^ observed unusual choroidal tissue beneath the excavation in some patients with CSC and discussed that the subsequent contraction of focal scarring of choroidal connective tissue may result in focal retraction of the RPE, leading to the formation of the FCE.

This study has several limitations. First, the number of eyes with iFCE was small. As the results show, most patients were asymptomatic, therefore, the chances of encountering patients with iFCE in clinical practice are low. Second, we could not exclude the possibility of patients having a history of eye diseases, which means that such cases should be grouped into the acquired FCE group. There may also be the possibility of developing chorioretinal diseases in the future. Thus, continuous observation may provide some clues on whether any specific clinical feature or symptoms could be predictors of some chorioretinal diseases.

In conclusion, we studied the clinical findings and visual function of patients with FCE, but without chorioretinal diseases. Despite the presence of morphological changes in the outer retina and choroid, most patients with iFCE showed no visual impairment, although some exhibited metamorphopsia. Further studies are needed to determine the clinical importance of iFCE.

## Methods

This retrospective observational case series was conducted at the Unoki Eye Clinic and Nippon Medical School. This study was approved by the Research Ethics Committee of the Graduate School of Medicine at the Nippon Medical School in Tokyo, Japan. This study adhered to the principles of the Declaration of Helsinki and informed consent was obtained from all the patients.

Patients were recruited from the Medical Retina Outpatient Clinic between January 2021 and March 2023. FCE was diagnosed based on the presence of focal excavation of the choroid without accompanying scleral ectasia or posterior staphyloma, as detected by spectral-domain OCT (Spectralis HRA-OCT; Heidelberg Engineering, Heidelberg, Germany) using horizontal macular volume scans, which were examined by two retinal specialists (A.O. and F.O.). We excluded eyes with any chorioretinal diseases, such as CSC, exudative/dry AMD, and uveitis; however, we included eyes with several drusen but without choroidal neovascularization, as confirmed by OCT angiography (Cirrus; Carl Zeiss Meditec, Dublin, CA, USA). We also excluded eyes with a history of chorioretinal disease, as identified by patients’ medical records or personal declarations.

All of the included patients underwent multimodal imaging, such as slit-lamp examination, fundus color photography, spectral-domain OCT, and FAF using blue light (λ = 488 nm). We measured the subfoveal choroidal thickness, defined as the distance between the line corresponding to Bruch’s membrane beneath the RPE and the chorioscleral interface, on enhanced depth imaging OCT images positioned at the center of the fovea, using a software caliper. When the excavation was located within the foveal center, it was measured using the distance between the supposed line of Bruch’s membrane and the chorioscleral interface. The maximum excavation diameter and depth were also measured.

Regarding visual function, we evaluated the BCVA using the Landolt C chart and expressed it as the logMAR and determined retinal sensitivity using the Humphrey Field Analyzer (HFA) 10–2 program (Carl Zeiss Meditec). Metamorphopsia severity was assessed using M-CHARTS (Inami Co., Tokyo, Japan) and Amsler grid testing (black grid on a white background). M-CHARTS consists of 19 double-dotted lines, and the dot spacing is at a visual angle of 0.2–2.0°. When a continuous line is substituted with a dotted line, and the dot interval is changed from fine to coarse, the line distortion decreases with increasing dot intervals until the line appears continuous. Both the vertical and horizontal meridians were assessed. The degree of aniseikonia was established using the NAT (Handaya, Tokyo, Japan). The test consisted of paired red and green semicircles with a target size of 4 cm, which allowed the measurement of the degree of aniseikonia from 1 to 24%. The two semicircles with different sizes in each pair were arranged in a series, wherein the difference varied in increments of 1%. Participants indicated a pair of semicircles that appeared to be of equal size. The actual size difference between the semicircles in the pair represents the degree of aniseikonia in the patient. We examined the participants at a distance of 40 cm in both the vertical and horizontal meridians, and used the mean values.

## Data Availability

The datasets used and/or analyzed during the current study are available from the corresponding author on reasonable request.
